# RETRACTION: SLC1A3 Promotes Gastric Cancer Progression via the PI3K/AKT Signalling Pathway

**DOI:** 10.1111/jcmm.18568

**Published:** 2024-08-07

## Abstract

**RETRACTION:** Xu L, Chen J, Jia L, Chen X, Moumin FA, Cai J. SLC1A3 Promotes Gastric Cancer Progression via the PI3K/AKT Signalling Pathway. *Journal of Cellular and Molecular Medicine* 2020;24(24):14392‐14404. https://doi.org/10.1111/jcmm.16060

The above article, published online on 3 November 2020 in Wiley Online Library (wileyonlinelibrary.com), has been retracted by agreement between the journal Editor‐in‐Chief, Stefan N. Constantinescu; the Foundation for Cellular and Molecular Medicine; and John Wiley & Sons Ltd. The retraction has been agreed due to concerns raised by third parties. Specifically, image elements of Figure 6G were found to have been previously published in a different scientific context.^1^ The corresponding author stated that a third‐party company partially generated the data presented in the article and suggested there may have been mishandling of the data. Additionally, the article mentions use of the unverifiable/unknown cell line GSE‐1 and presents results from the cell line MKN‐28, reported as misidentified by the Japanese Collection of Research Bioresources (JCRB) Cell Bank of the National Institutes of Biomedical Innovation, Health and Nutrition (https://cellbank.nibiohn.go.jp/). Therefore, the editors consider the conclusions of this article to be invalid. The authors have been informed of the decision of retraction.

[1] Pang X, Li R, Shi D, Pan X, Ma C, Zhang G, Mu C, Chen W. Knockdown of Rhotekin 2 expression suppresses proliferation and induces apoptosis in colon cancer cells. *Oncol Lett*. 2017;14(6):8028‐8034. https://doi.org/10.1111/jcmm.16060

**RETRACTION:** Xu L, Chen J, Jia L, Chen X, Moumin FA, Cai J. SLC1A3 Promotes Gastric Cancer Progression via the PI3K/AKT Signalling Pathway. *Journal of Cellular and Molecular Medicine* 2020;24(24):14392‐14404. https://doi.org/10.1111/jcmm.16060

The above article, published online on 3 November 2020 in Wiley Online Library (wileyonlinelibrary.com), has been retracted by agreement between the journal Editor‐in‐Chief, Stefan N. Constantinescu; the Foundation for Cellular and Molecular Medicine; and John Wiley & Sons Ltd. The retraction has been agreed due to concerns raised by third parties. Specifically, image elements of Figure 6G were found to have been previously published in a different scientific context.^1^ The corresponding author stated that a third‐party company partially generated the data presented in the article and suggested there may have been mishandling of the data. Additionally, the article mentions use of the unverifiable/unknown cell line GSE‐1 and presents results from the cell line MKN‐28, reported as misidentified by the Japanese Collection of Research Bioresources (JCRB) Cell Bank of the National Institutes of Biomedical Innovation, Health and Nutrition (https://cellbank.nibiohn.go.jp/). Therefore, the editors consider the conclusions of this article to be invalid. The authors have been informed of the decision of retraction.

[1] Pang X, Li R, Shi D, Pan X, Ma C, Zhang G, Mu C, Chen W. Knockdown of Rhotekin 2 expression suppresses proliferation and induces apoptosis in colon cancer cells. *Oncol Lett*. 2017;14(6):8028‐8034. https://doi.org/10.1111/jcmm.16060

